# Orally administered *Lactobacillus casei* exhibited several probiotic properties in artificially suckling rabbits

**DOI:** 10.5713/ajas.18.0973

**Published:** 2019-04-15

**Authors:** Xue Mei Shen, Hong Xiao Cui, Xiu Rong Xu

**Affiliations:** 1 College of Animal Science and Technology, Northwest A&F University, Yangling, Shaanxi, 712100, China

**Keywords:** Degranulation, Inflammatory Factors, Lysozyme, Paneth Cells, Vermiform Appendix

## Abstract

**Objective:**

*Lactobacilli* in rabbit intestine is rare and its function in rabbit gut health is not fully understood. The present study aimed to evaluate *in vivo* the probiotic potential of *Lactobacillus casei* for suckling rabbits.

**Methods:**

Two healthy 5-day-old suckling rabbits with similar weights from each of 12 New Zealand White litters were selected and disturbed to control group and treatment group. All rabbits were artificially fed. The treatment group had been supplemented with live *Lactobacillus casei* in the milk from the beginning of the trial to 13 days of age. At 15 days of age, healthy paired rabbits were slaughtered to collect intestinal samples.

**Results:**

i) Oral administration of *Lactobacillus casei* significantly increased the proportion of *Lactobacilli* in the total intestinal bacteria (p<0.01) and obviously reduced that of *Escherichia-Shigella* (p<0.01); ii) treatment increased the length of vermiform appendix (p<0.05); iii) a higher percentage of degranulated paneth cells was observed in the duodenum and jejunum when rabbits administered with *Lactobacillus casei* (p<0.01); and iv) the expression of toll-like receptor 9, lysozyme (*LYZ*), and defensin-7-like (*DEFEN*) in the duodenum and jejunum was stimulated by supplemented *Lactobacillus casei* (p<0.05).

**Conclusion:**

Orally administered *Lactobacillus casei* could increase the abundance of intestinal *Lactobacilli*, decrease the relative abundance of intestinal *Escherichia-Shigella*, promote the growth of appendix vermiform, stimulate the degranulation of paneth cells and induce the expression of *DEFEN* and *LYS*. The results of the present study implied that *Lactobacillus casei* exhibited probiotic potential for suckling rabbits.

## INTRODUCTION

Rabbits, especially suckling rabbits and newly weaned rabbits, are susceptible to intestinal infection, which often leads to a serious inflammatory response and death [1,2]. Inclusion of antibiotic in diet is the main strategy to prevent rabbit from pathogenic infection. However, rabbit is herbivore with big cecum, antibiotic supplementation to diet can inhibit cecal fermentation, and thus decrease rabbit’s feed efficiency and growth performance [3]. As the alternative to dietary antibiotics, probiotics have been widely used to prevent intestinal pathogenic infection, and *Lactobacilli* are the most popular commercial probiotics for different animals. However, Yu and Tsen [4] once reported that the lack of adhesive capability prevented *Lactobacilli* from colonizing in the intestinal tract of rabbit, and *Lactobacilli* is rare in any part of rabbit’s gastrointestinal tract [5,6]. Therefore, the use of *Lactobacilli* in rabbit’s diet is debated. The present study aimed to investigate whether *Lactobacilli* is an efficient probiotic for rabbits. Generally, probiotic properties of a bacterium to the host are evaluated *in vitro* by testing its antimicrobial potential [7], adhesion ability to the host’s intestinal mucin [8] and resistance to the gastrointestinal environment [9], while the *in vivo* evaluation of their other probiotic properties, such as the ability to stimulate the development of intestinal immune system and the ability to regulate intestinal innate immune and inflammation homeostasis, is ignored [10].

The vermiform appendix is a unique and important intestinal immunity organ for rabbit [11]. It is well known that the development of intestinal immune system is stimulated by commensal bacteria. Previous research showed that components from *Bacteroides fragilis* and *Bacillus subtilis* could promote the development of rabbit vermiform appendix, but those from other investigated bacteria, such as *Clostridium subterminale* and *epidermidis*, could not [11]. This suggested that the development of rabbit vermiform appendix is stimulated only by specific commensals, but it is unclear whether *Lactobacilli* belongs to these specific commensals.

Paneth cells are important innate immune cells. Evidence has proven that they play an important role in protecting the small intestine against pathogenic infection and in regulating intestinal microbial density by releasing antimicrobial α-defensin, lysozyme, peptides, and secretory phospholipase A2 [12]. Regulation of the secretory function in paneth cells is also involved in the physiological interactions between commensal bacteria and their host. For example, degranulation in mouse paneth cells has been observed after mice were orally treated with both live bacteria and killed bacteria [13,14]. The results of these studies suggested that an important property of probiotics is their role in regulating intestinal antimicrobial activity of the host.

The intestinal inflammatory response is also involved in the interaction of commensal bacteria and their host. Our recent study showed that total intestinal bacteria from rabbit tends to induce a higher inflammatory level in cultured crypt and villus of rabbit than total intestinal bacteria from chicken or pig [15], probably because of the low abundance of *Lactobacilli* in rabbit’s intestine. It was reported that intestinal dsRNA can alleviate intestinal inflammation, and *Lactobacilli* have much higher level of dsRNA than other investigated bacteria [16]. Therefore, the possible function of orally administered *Lactobacillus casei* in mediating the inflammatory level in rabbit’s intestine is worth to be investigated.

For the above-mentioned reasons, the present study was conducted to evaluate *in vivo* the probiotic potential of *Lactobacillus casei* (one of the widely used *Lactobacillus* probiotics) for suckling rabbits by investigating the effects of *Lactobacillus casei RABX*1, which was previously isolated from the rabbit intestine in our laboratory and selected based on the evaluation *in vitro* for probiotic properties, including adhesion ability to rabbit intestinal mucin, resistance to the gastric acid and intestinal cholate, and antibacterial ability to *Escherichia coli* [17]. Its effects on the development of the appendix vermiform, degranulation of paneth cells, expression of defensin-7-like (*DEFEN*) and lysozyme (*LYZ*), and the inflammatory response in artificially suckling rabbits were investigated in present study.

## MATERIALS AND METHODS

### Animal care

All animal protocols were pre-approved by the Animal Protection Committee of Northwest A&F University, and the use of animals and the experimental procedure was consulted to the Guide for the Care and Use of Laboratory Animals of NIH.

### Selection of suckling rabbits and feeding experiment

Twelve newborn litters of New Zealand White were selected based on doe’s parity and health. Two healthy male kits with similar body weights were selected from each of these 12 litters at 5-day-old and then randomly distributed into the control group and treatment group. The body weight difference between the two kits in the same pair is <2.0 g at 5 days of age. All the selected kits were artificially fed with milk from the beginning to the end of the trial. All the kits were fed five times (8:00 am, 12:00 am, 4:00 pm, 8:00 pm, and 12:00 pm) every day with a commercial milk powder (without probiotic in the powder) for pet infant. The milk powder was dissolved in warm (37°C to 38°C) boiled water (milk powder:water = 1 g:4 mL) before feeding. The volume of the dissolved milk fed to each kit each time was 5 mL (5 d), 8 mL (6 d), 10 mL (7 to 8 d), 15 mL (9 to 11 d), 20 mL (12 to 13 d), and 25 mL (14 to 15 d). Kits in the treatment group had been continuously supplemented with *Lactobacillus casei RABX*1 (accession number: KT944253) from 5 days of age to 13 days of age. To prevent the interference from the supplemented *Lactobacilli*, which would affect the investigated percentage of intestinal *Lactobacilli* in total bacteria, kits in the treatment group fed milk without *Lactobacillus casei RABX*1 from 14 days of age. Before being added to the milk, the isolated bacterium was cultured in sterile Mann-Rogosa-Sharp broth at 37°C for 12 h and harvested by centrifugation at 5,000 g for 6 min and then washed with sterile phosphate buffered saline. The number of the harvested bacteria was estimated by a spectrophotometer. Bacteria were then resuspended in the milk to reach a concentration of 5–6×10^8^ colony-forming units/mL and orally administered to kits in the treatment group. All the kits were caged in boxes with constant temperature of 30°C (moisture 50% to 70%). The kits of the same group were caged in the same box with natural lighting. The padding towels were changed twice every day and sterilized in boiled water after being washed, and the boxes were cleaned three time every day (before feeding). All the kits were weighed at 5 days and 15 days of age before morning feeding, and their health condition was recorded every day, the unhealthy kits were separated from its group as soon as their abnormality were found.

### Sampling and measurement of vermiform appendix length

Healthy pairs of kits were slaughtered eight hours after the morning feeding at 15 days of age. Before sampling, the length of the vermiform appendix was quickly measured from its end to the junction between the cecum and vermiform appendix. Then, the content in small intestine was collected and mixed together to determine the relative proportion of intestinal *Lactobacilli* or *Escherichia-Shigella* in total bacteria (Note: the content in kit’s duodenum, jejunum and ileum wasn’t collected separately because it was too less). Two parts each were harvested from the duodenum, jejunum and ileum. One part was stored at −80°C for determination of gene expression, and another part was fixed in 4% paraformaldehyde for investigation of the intestinal morphology and degranulation of paneth cell.

### Determination of the relative proportion of ileal *Lactobacilli* and *Escherichia-Shigella* in total bacteria

Total bacterial genomic DNA in the collected intestinal content was extracted by the modified phenol-chloroform-isoamylalcohol extraction method [18]. The concentration of the extracted DNA solution was subsequently determined and then diluted to 15 ng/μL. The relative proportion of *Lactobacilli* or *Escherichia-Shigella* in total bacteria was determined by relative quantification real-time polymerase chain reaction (PCR). The reaction mixture (20 μL) consisted of 10 μL of SYBR Premix Ex Taq (Takara Biotechnology [Dalian] Co., Ltd, Dalian, Liaoning, China), 0.4 μM of each primer and 30 ng of the extracted bacterial genomic DNA. The average cycle threshold (Ct) value for *Lactobacilli*, *Escherichia-Shigella*, or total bacteria in each sample was determined in triplicate. Then, the average Ct value of *Lactobacilli* (or *Escherichia-Shigella*) after being normalized to that of total bacteria was used for calculating the relative proportion of *Lactobacilli* (or *Escherichia-Shigella*) in total bacteria by using the 2^−ΔΔCt^ method as previously described [18]. Real-time PCR was performed on the IQ5 Cycler (Bio-Rad Laboratories, Inc., Hercules, CA, USA). The primers used for detecting 16S rDNA of *Escherichia-Shigella*, *Lactobacilli* and total bacteria are listed in Table 1.

### Measurement of intestine morphology and quantitation of paneth cell degranulation

The fixed intestine sections were rinsed with water and then dehydrated in a graded series of ethanol. After being cleared in benzene twice, sections were saturated with paraffin first and then embedded in paraffin. The embedded sections were cut into thin slices (5 μm thickness, 10 slices of each sample), which were stained with hematoxylin/eosin and prepared for observation by light microscopy. A total of 10 intact, well-oriented crypt-villus units were selected in triplicate for each intestinal cross-section. Villus height was measured from the tip of the villus to the villus-crypt junction. Crypt depth was defined as the depth of the invagination between two adjacent villi. The intestine morphological measurements were performed by using an image processing and analysis system (Optimus software version 6.5, Media Cybergenetics, North Reading, MA, USA). Quantitation of degranulated paneth cells was performed following the method described by Rumio et al [14]. The fixed intestinal sections were embedded in paraffin and then cut into 5-μm slices. After staining with hematoxylin and eosin, the slices were mounted in Entellan (Merck, Darmstadt, Germany) and then observed with a ViCo microscope (Biomedica Mangoni S.n.C., Nikon Instruments S.p.A, Pisa, Italy) equipped with a digital Nikon DS-L1 camera. Paneth cells were quantitated only in crypts that were sectioned through their center and could present a clear lumen. Degranulated paneth cells were distinguished from non-degranulated paneth cells based on the increased granule dimension and presence of large darkly colored vacuoles in the cytoplasm [14]. The percentage of degranulated paneth cells in each crypt was calculated, and twenty crypts were analyzed for each intestinal segment.

### Determination of gene expression by quantitative real-time polymerase chain reaction

Total RNA of each intestine sample stored at −80°C was extracted using Trizol reagent (Invitrogen, Carlsbad, CA, USA). The cDNA was synthesized from 1 μg of total RNA using a reverse transcriptase kit (Takara Biotechnology [Dalian] Co., Ltd, China). The relative mRNA levels of toll-like receptor 9 (*TLR9*), *DEFEN*, *LYZ*, tumor necrosis factor alpha (*TNF-α*), interferon beta (*IFN-β*), and interleukin 6 (*IL-6*) in the intestine were quantified by real-time quantitative PCR, which was carried out on the IQ5 Cycler using the TaKaRa SYBR Premix Ex Taq kit (China). The reaction system contained 1 μL of synthesized cDNA, 1 μL of each primer (4 μM), 10 μL of SYBR Premix Ex Taq (Takara Biotechnology [Dalian] Co., Ltd, China) and 7 μL of nuclease-free water. The cycle threshold (Ct) value for the investigated gene in each sample was determined in triplicate, and the average Ct value was calculated. Finally, the average Ct values for each gene, after being normalized to that of glyceraldehyde-3-phosphate dehydrogenase gene, were used for quantification by the 2^−ΔΔCt^ method. Real-time PCR was carried out with an initial denaturation step of 95°C for 5 min, followed by 40 cycles of 95°C for 15 s and 60°C for 45 s. The primers designed using Primer 5.0 or referenced are shown in Table 2.

### Statistics

The data collected from the experiment were analyzed with a paired-samples *t* test using SPSS19.0 soft package (SPSS Inc, Chicago, IL, USA), and the results were presented as mean± standard deviation for each trait. The probability of significance is indicated by the following conventional standard abbreviations: p>0.05 for non-significance and p<0.05, p<0.01, and p<0.001 for significance at these levels.

## RESULTS

During the feeding experiment, two kits in control group and three in treatment group died at different time, all the dead kits came from the pairs with lighter initial body weight, and they died of diarrhea (one in control, one in treatment) or no symptoms (one in control, two in treatment). One dead kit in control group and one dead kit in treatment group came from the same litter therefore, all the following investigated data were collected from eight healthy kit pairs. There was no difference in average daily gain between the two groups (p = 0.759) (data not shown).

### Effect of treatment on the proportion of *Lactobacilli* or *Escherichia-Shigella* in total bacteria

The relative proportion of the investigated bacterium in total bacteria was measured by real-time PCR. As presented in Table 3, although the oral administration of *Lactobacillus casei* had been stopped for more than 24 h, suckling rabbits in the treatment group had an extremely higher relative proportion of *Lactobacilli* in total intestinal bacteria (p<0.001) and an extremely lower relative proportion of *Escherichia-Shigella* (p< 0.001) than rabbits in the control group.

### Effect of treatment on intestinal morphology, vermiform appendix length and percentage of degranulated paneth cells

**Figure 1:**
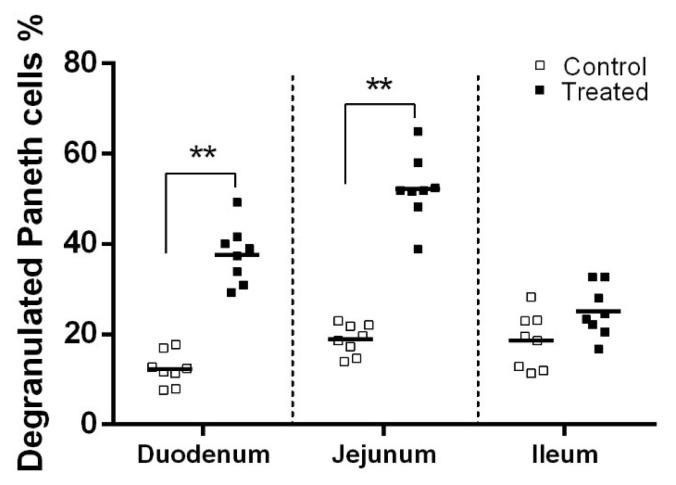
Effect of orally administered *Lactobacillus casei* (treated) on the percentage of degranulated paneth cells in the intestine of suckling rabbits.

Oral administration with *Lactobacillus casei* did not change the intestinal morphological indices, including villus height, crypt depth and the ratio of villus height to crypt depth (p> 0.05), but it significantly increased the length of the vermiform appendix in suckling rabbits (p = 0.04) (Table 4). Degranulated paneth cell was showed in Supplementary Figure S1. As presented in Figure 1, the percentage of degranulated paneth cells in the duodenum and jejunum of suckling rabbits orally administered with *Lactobacillus casei RABX*1 was increased by 206% and 177% (p<0.001), respectively. Degranulation of paneth cells was also observed in ileum sections, but the difference in the percentage of degranulated paneth cells between the two groups was not obvious (19%±6.1% in the control group vs 25%±5.7% in the treatment group).

### Effect of oral administration with *Lactobacillus casei* on expression level of the investigated genes

**Figure 2:**
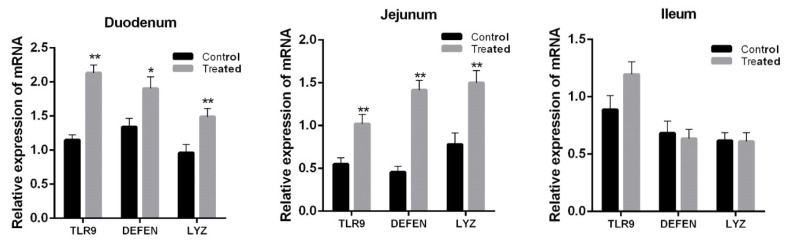
Effect of orally administered *Lactobacillus casei* (treated) on the relative expression of *TLR9* and *LYZ* in the intestine of suckling rabbits. *TLR9*, toll-like receptor 9; *LYZ*, lysozyme.

**Figure 3:**
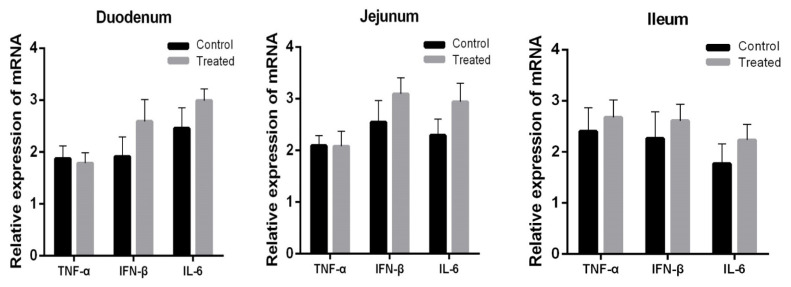
Effect of orally administered *Lactobacillus casei* (treated) on the relative expression of *TNF-α*, *IFN-β*, and *IL-6* in the intestine of suckling rabbits. *TNF-α*, tumor necrosis factor alpha; *IFN-β*, interferon beta; *IL-6*, interleukin 6.

Expression of *TLR9*, *DEFEN*, and *LYZ* in the duodenum (p< 0.001, p = 0.05, and p = 0.008, respectively) and jejunum (p< 0.001, p<0.001, and p = 0.001, respectively) was significantly increased by orally administered *Lactobacillus casei RABX*1 (Figure 2), but that in the ileum was not affected (p>0.05). Oral administration with *Lactobacillus casei* did not change the expression level of *TNF-α*, *IFN-β*, and *IL-6* in the duodenum, jejunum and ileum of artificially suckling rabbits (Figure 3).

## DISCUSSION

Probiotic properties have bacterium-host specificity. Unlike in the intestines of many animals such as chicken, the dominant genus in the intestine of rabbit is not *Lactobacilli*, which occupies less than 1% of the total intestinal bacteria in rabbit [19]. It is necessary to confirm whether *Lactobacilli* can be used as an effective probiotic for rabbits. Therefore, the present study explored *in vivo* the probiotic properties of *Lactobacillus casei* in suckling rabbit. The development of intestinal microflora begins at the day of birth and is affected by many factors [23], including the bacterial structures in the mother’s birth canal or milk, so we designed a paired experiment and artificially fed suckling rabbits from the fifth day after their birth to minimize the influence from their mother. Kits were not separated from their mother before 5 days old because we found that kits could not survive if they did not suck their mother’s colostrum.

Considering the strain specificity effect of probiotics, we evaluated *in vitro* the adhesion ability to rabbit intestinal mucin and resistance to the gastrointestinal environment of several *Lactobacilli* species, and the strain *Lactobacillus casei RABX*1 exhibited good properties for the *in vitro* evaluated indices. To make sure that the investigated *Lactobacilli* was not the administered *Lactobacillus casei RABX*1 themselves, administration of *Lactobacillus casei RABX*1 in treatment group was stopped after 13 days of age. The fact that the relative proportion of *Lactobacilli* in total bacteria (detected after the administration of *Lactobacillus casei RABX*1 had been stopped for about 40 h) in the treatment group extremely higher than that in the control group indicated that *Lactobacillus casei RABX*1 could effectively get to the small intestine of artificially suckling rabbits and be proliferous there. Probiotics can suppress pathogens through different mechanisms [24], including competition with pathogens for nutrients and inhibition of pathogens by producing antimicrobial metabolites. The decrease in *Escherichia-Shigella* in the intestine of suckling rabbit should be related to the suppressive function of increased intestinal *Lactobacilli*.

The development of the vermiform appendix is associated with the immune capacity of the intestine of a rabbit. Štěpánková et al [25] reported that normal rabbit had a much better developed vermiform appendix than germ-free rabbit, and our recent study found that suckling rabbits with more opportunity to contact with their mother had more intestinal commensal bacteria and longer vermiform appendices [26]. These two studies indicated that the development of the special intestinal immune organ in rabbit needs bacterial stimulation. Rhee et al [11] proved that it was special commensals such as *Bacteroides fragilis* and *Bacillus subtilis* that could stimulate the development of the vermiform appendix. The present study showed that orally administered *Lactobacillus casei* also promoted the development of the vermiform appendix in suckling rabbit.

Degranulation is the way that a paneth cell secretes its synthesized antimicrobial substances, such as defensin and *LYZ*, to the intestinal lumen [14]. The action of degranulation is regulated by the *TLR9* signaling pathway, which can be stimulated by special fragments of bacterial DNA [27]. The increased percentage of degranulated paneth cells and expression levels of *TLR9*, *DEFEN*, and *LYZ* in the duodenum and jejunum in the present study indicated that exogenous *Lactobacillus casei* was involved in the regulation of paneth cell function in suckling rabbit and suggested another probiotic property of *Lactobacillus casei* for rabbits. The expression of *TLR9*, *DEFEN*, and *LYZ* in the ileum was not affected by treatment, which was inconsistent with the duodenum and jejunum. This inconsistency was probably derived from the uneven distribution of paneth cells in different intestinal segments. It was reported that most paneth cells locate in the forepart of the small intestine [12], and our morphological test also showed that paneth cells were rare in the rabbit ileum.

Components of commensal bacteria can alleviate intestinal inflammation by regulating the expression of pro-inflammatory factors and anti-inflammatory factors. Kawashima et al [16] reported that bacterial double-stranded RNA (dsRNA) showed a regulatory function by triggering anti-inflammatory factor *IFN-β* production and inhibiting pro-inflammatory factor production, and they also proved that *Lactobacilli* contains higher dsRNA than other investigated bacteria. Furthermore, it was reported that *TLR9* signaling, which was stimulated by orally administered *Lactobacillus casei* in the intestine of suckling rabbit in present study, had an anti-inflammatory effect on murine experimental colitis [28]. However, increased intestinal *Lactobacilli* here neither induced higher expression of the anti-inflammatory factor *IFN-β* nor inhibited that of pro-inflammatory factors. It meant that the combined effect of the supplemented *Lactobacillus casei* did not alter inflammation homeostasis in the intestine of healthy suckling rabbits. Subsequent research should investigate the probiotic effect of *Lactobacillus casei* supplementation on the anti-inflammatory response in suckling or newly weaned rabbits when they are suffering from intestinal inflammation.

In conclusion, orally administrated *Lactobacillus casei* decreased the relative proportion of *Escherichia-Shigella* in total intestinal bacteria, increased the relative proportion of *Lactobacilli* in total intestinal bacteria, stimulated development of the appendix vermiform, and induced degranulation of paneth cells and the expression of *TLR9* and *LYZ* in suckling rabbit. In conclusion, orally administered *Lactobacillus casei* could effectively increase the abundance of intestinal *Lactobacilli* and exhibit several probiotic properties for suckling rabbit. However, it didn’t improve the growth performance during the experimental period. Further study should be conducted to investigate the subsequent positive effect of early administrated *Lactobacillus casei* on newly weaned and/or growing rabbit.

## Figures and Tables

**Table 1:** The specific primers for bacteria

Items	Primer	Primer sequence (5′-3′)	Amplicon size (bp)	References
Total bacteria	Forward	ACTCCTACGGGAGGCAGCAGT	174	[19]
	Reverse	ATTACCGCGCTGCTGGC		
*Lactobacilli*	Forward	CACCGCTACACATGGAG	197	[20]
	Reverse	AGCAGTAGGGAATCTTCCA		
*Escherichia-Shigella*	Forward	CATGCCGCGTGTATGAAGAA	96	[21]
	Reverse	CGGGTAACGTCAATGAGCAAA		

**Table 2:** The gene-specific primer pairs for real-time polymerase chain reaction analysis

Gene	Sequence (5′-3′)	Product size (bp)	GenBank accession No.
*GAPDH*	F: AGAGCACCAGAGGAGGACG	104	NM_001082253.1 [22]
	R: TGGGATGGAAACTGTGAAGAG		
*TLR9*	F: AGAAGTCGTCCTTTGCCCAG	180	XM_008260758.2
	R: AGGCCTGGGTGATGAAGTTG		
*LYZ*	F: GCCGCTACTGGTGTAACGAT	126	XM_002711323.3
	R: GATCGCTGACGACCCTCTTT		
*DEFEN*	F: ATGTGGTTCCAGACCAGGAG	165	XM_017339330.1
	R: TGCAGGTGCCATAGATGTGT		
*TNF-α*	F: CGTAGTAGCAAACCCGCAAGTG	152	NM_001082263.1
	R: CGCTGAAGAGAACCTGGGAGTAG		
*IFN-β*	F: TCCAACTATGGCACGGAAGTCT	133	XM_002707968.4
	R: TTCTGGAGCTGTTGTGGTTCCT		
*IL-6*	F: CTACCGCTTTCCCCACTTCAG	135	NM_001082064.2
	R: TCCTCAGCTCCTTGATGGTCTC		

*GAPDH*, glyceraldehyde-3-phosphate dehydrogenase; *TLR9*, toll-like receptor 9; *LYZ*, lysozyme; *DEFEN*, defensin-7-like; *TNF-α*, tumor necrosis factor alpha; *IFN-β*, interferon beta; *IL-6*, interleukin 6.

**Table 3:** Effect of treatment on the relative proportion of *lactobacillus* and *Escherichia-Shigella* in total bacteria

Item	Control	Treatment	p-value
*Lactobacillus*	0.90±0.14	116.14±19.46	<0.001
*Escherichia-Shigella*	30.37±4.60	1.20±0.22	<0.001

**Table 4:** Effects of *Lactobacillus casei* on intestinal morphology and appendix length of suckling rabbits

Items	Control	Treatment	p-value
Villus height (μm)			
Duodenum	384.49±25.91	365.19±28.41	0.668
Jejunum	386.23±16.03	396.17±17.75	0.703
Ileum	369.73±17.99	337.61±31.34	0.427
Crypt depth (μm)			
Duodenum	68.10±4.16	67.82±2.41	0.962
Jejunum	63.32±2.87	63.36±2.50	0.990
Ileum	50.73±2.51	50.38±3.71	0.924
V/C			
Duodenum	5.71±0.34	5.40±0.41	0.612
Jejunum	6.22±0.44	6.28±0.28	0.903
Ileum	7.37±0.43	6.86±0.70	0.528
Vermiform appendix length (cm)	4.22±0.18	4.96±0.33	0.044

V/C, villus height/crypt depth.
