# Supplementary material for: Orally administered Lactobacillus casei exhibited several probiotic properties in artificially suckling rabbits
Source: Asian-Australas J Anim Sci. 2019 Apr 15;33(8):1352–9. doi: 10.5713/ajas.18.0973 (PMC7322641; doi:10.5713/ajas.18.0973)
Supplement: Supplementary file 1 [file ajas-18-0973-suppl1.pdf]

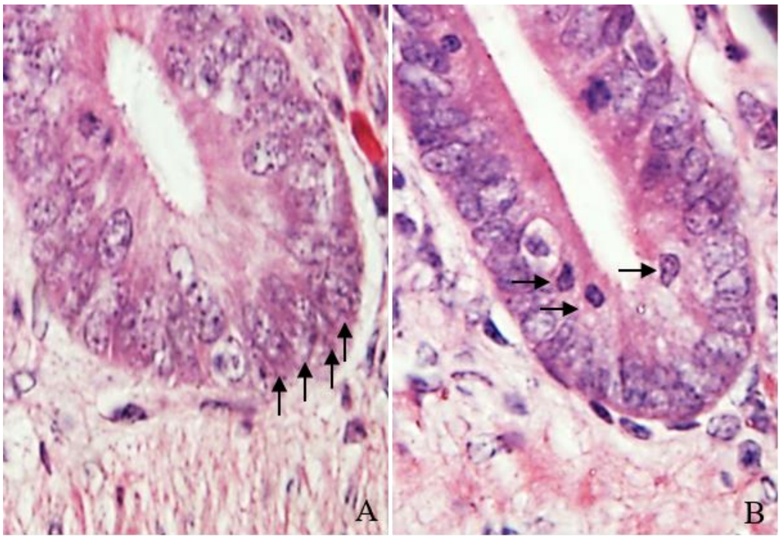

Supplementary Figure S1. Morphology of intestinal paneth cell and its degranulation (×1000)

Notes: The H&E stained crypts in jejunum of suckling rabbit. Arrowhead on A shows paneth cell that is not in state of degranulation, while arrowhead on B shows paneth cells that is in state of degranulation with clear large dark-colored vacuoles in their cytoplasm.
